# Comparison between anterolateral thigh perforator free flaps and pectoralis major pedicled flap for reconstruction in oral cancer patients-A quality of life analysis

**DOI:** 10.4317/medoral.19276

**Published:** 2013-10-13

**Authors:** Yan Xiao, Juanfang Zhu, Xiangping Cai, Jing Wang, Fei Liu, Haibin Wang

**Affiliations:** 1 MD, PHD. Department of Stomatolagy, the First Affiliated Hospital of Zhengzhou University, Zhengzhou, Henan 450052, China; 2 MD PHD. Department of Stomatolagy, the Third People’Hospital of zhengzhou City , Zhengzhou, Henan 450052, China

## Abstract

The aim of this study was to compare the differences between anterolateral thigh perforator free flaps (ALTFF) and pectoralis major myocutaneous flap (PMMF) for reconstruction in oral cancer patients. Method and Patients: who received free flap or PMMF reconstruction after ablation surgeries were eligible for the current study. The patients’ demographic data, medical history, and quality of life scores(Medical Outcomes Study-Short Form-36 (MOS SF-36) and the University of Washington Quality of Life (UW-QOL) questionnaires were collected. Results: 81 of 118 questionnaires were returned (68.64%). There was signiﬁcant differences between two groups in the gender (P<0.005). Patients reconstructed with ALTFF had better appearance domains and better shoulders domains, in addition to better role emotion domains. Conclusions: Using either PMMF or ALTFF for reconstruction of oral defects after cancer resection signiﬁcantly inﬂuences a patient’s quality of life. Data from this study provide useful information for physicians and patients during their discussion of reconstruction modalities for oral cancers.

** Key words:**Quality of life, ALTFF,PMMF, oral cancer.

## Introduction

The reconstruction oral cavity defects represents a challenge because of the critical role of this area both esthetically and functionally. In the past, attempts were made to achieve functional restoration of resected head and neck areas with acceptable cosmesis using local and locoregional flaps. The pectoralis major myocutaneous flap (PMMF), based on the thoracoacromial artery, was described in 1979 by Ariyan (1). PMMF is well established as one of the most important reconstructive methods in major oral cancer surgery due to its simple technical aspects, versatility, and proximity to the oral cavity region (2).

Presently, free flap techniques represented a revolution in reconstructive surgery as they enabled the harvesting of a large amount of revascularized tissue; it could be tailored to the defect and allowed for more complex reconstructive procedures, while simultaneously permitting more extensive head and neck resections (3).So free flap was the favored method for reconstruction after major oral cancer surgery.For free flaps, the ALTFF have proven to be very reliable and an average flap survival rate of 95% is usually achieved in experienced hands (4).The ALTFF was first reported by Song in 1984, it has gained popularity in oral cavity reconstructions. It has some advantages, including a long pedicle with a suitable vessel diameter, the availability of different tissues with large amounts of skin, and its adaptability as a sensate or flow-through flap if necessary (5).

Although the major intended outcome of oral cavity cancer surgery is still the survival of the patient, quality of life is now seen as an essential secondary outcome. Assessment of the quality of life (QOL) provides information about the psychosocial well-being of patients and the effects of the disease and its treatment. Hence, it is an important tool for evaluating outcome in conjunction with mortality, morbidity, survival and recurrence rates. In addition, few studies have evaluated the differences in quality of life between patients with oral cavity cancers reconstructed with PMMF compared with those who underwent ALTFF. Therefore, the aim of this study was to compare the differences between PMMF and ALTFF for the reconstruction of the oral cavity defect in oral cancer patients.

## Material and Methods

-Patients

Patients for this study had reconstructive surgery in the period 2004-2011, in the Department of Stomatolagy, First Affiliated Hospital of Zhengzhou University. Once patients had been diagnosed with oral cancer, they accepted the immediate reconstruction with PMMF or ALTFF. Because this study was retrospective it was granted an exemption in writing by in the First Affiliated Hospital of Zhengzhou University of Ethical Review Board. In this study, the inclusion criteria were: the flap survived completely; the patient’s age was less than 70 years; patients had no previous or synchronous malignancies; patients had no cognitive impairment; QOL was assessed at least one years after reconstruction. Patients with recurrence of the disease were not excluded from the study. 118 patients (93 male, 25 female) met the inclusion criteria. Most patients completed the questionnaire when they returned to the hospital for their regular compliance review. The remaining patients received a formal letter explaining the study, an informed consent form and the Medical Outcomes Study-Short Form-36 (MOS SF-36)/UW-QOL questionnaire. Those patients who did not reply within four weeks received a reminder. Patient characteristics are summarized in  Table 1.

**Table 1:**
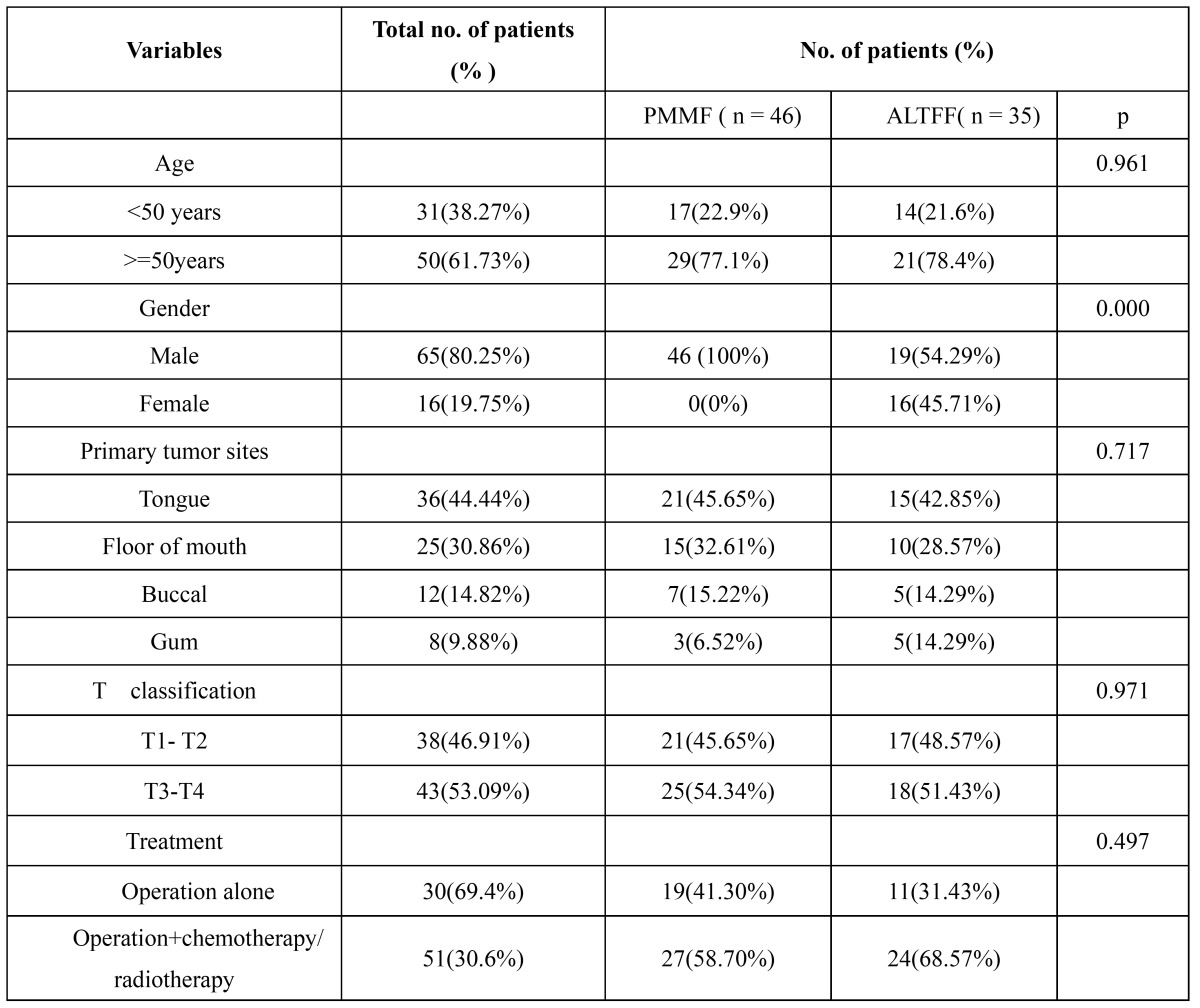
Clinical data analyses of oral cavity cancer patients who underwent PMMF or ALTFF for reconstruction.

-Questionnaires and data collection

Although many generic QOL measurements have been developed over the past 30 years, the Medical Outcomes Study-Short Form-36 (MOS SF-36) (6) and the University of Washington Quality of Life (UW-QOL) (7) are the two that are most commonly used for cancer patients. The MOS SF-36 questionnaire comprises 36 items that fall into eight health domains: physical functioning, role physical, bodily pain, general health, vitality, social functioning, role emotion, and mental health. Scores can range from 0 (worst) to 100 (best). The standard MOS SF-36 is available as a Chinese version and has been validated for a Chinese population (8).

The most recently modified version of the University of Washington Quality of Life (UW-QOL) questionnaire was used in this study. This questionnaire evaluates the functional outcome of patients who underwent vascularized free forearm flap and free anterolateral thigh perforator flap reconstruction. The questionnaire is composed of 15 domains: 12 are disease-specific items (pain, appearance, activity, recreation, swallowing, chewing, speech, shoulder, taste, saliva, mood, and anxiety), and 3 are global questions. Each of the 12 included questions has 3-6 response options. The domains are scored on a scale ranging from 0 (worst) to 100 (best).

Besides the 15 questions, patients were asked to choose no more than 3 of the 12 disease-specific domains that had been the most important to them in the preceding 7 days. We scored the individual domains according to the UW-QOL guidelines. The standard UW-QOL is available as a Chinese version and has been validated for a Chinese population (9).

-Statistical analysis

Data were recorded and analyzed with SPSS 16.0 statistical software. Comparisons of nominal or ordinal variables between patients who underwent surgery with the PMMF and ALTFF was analyzed using a chi-square test or Fisher’s exact test. The SF-36 and UW-QOL scores were compared for each domain using the nonparametric Mann-Whitney tests. The significance level was set to p < 0.05.

## Results

From 2004 to 2011, a total of 118 patients with oral cavity cancer underwent ablation surgery followed by either PMMF or ALTFF reconstruction. 46 patients (38.98%) received ALTFF whereas 72 patients (61.02%) received PMMF reconstruction. Of the 118 questionnaires, 81 were returned. Of the 81 patients who completed the questionnaires, there were 65 men and 16 women with a median age of 53.6 (range 24 - 70). 35 of the 81 patients had received ALTFF, and 46 patients underwent PMMF. The tongue (N=36,44.44%) and floor of mouth (N=25,30.86%) were the most common sites ( Table 1). Followed by buccal (N=12,14.82%), and gum (N=8, 9.88%).38 patients (46.91%) located in T1-T2 classification, while 43 patients (53.09%) located in T3-T4 ( Table 1).

The postoperative follow-up period ranged from 12 months to 8 years, and the mean follow-up point was 3.5 years. A number of patients did not participate further in the study due to a variety of reasons: There were 15 returned informing us that the patients had died and 10 questionnaires returned stating that the patients had changed their address, 8 patients refused to participate; 3 patients refused to sign the informed consent form; 1 patients claimed not to have received the questionnaires or the reminder.

There were no significant statistical difference between the PMMF and ALTFF groups in age (p=0.961), primary tumor site (p=0.717), T-stage (p=0.971) and treatment (p=0.497). However, all of female patients received ALTFF than did male patients (P<0.005). Furthermore, there was significant difference between the PMMF and ALTFF groups in operation time (475 ± 128 vs 565 ± 107 min).

There were also no significant differences between the two groups for the pain, activity, recreation, swallowing, chewing, speech, taste, saliva, mood and anxiety domains. However, there were significant differences between the PMMF and ALTFF groups for the shoulder (64.15±9.80vs69.57±17.53,p=0.009) and appearance domains (58.52 ±12.83 vs 67.37±9.42, p=0.001) ( Table 2). When patients were asked to select their three most important domains, speech was considered the most important over the past 7 days, followed by chewing, swallowing and saliva. Pain domains were considered the least important to patients.

There was a significant difference for the role emotion (65.59± 9.91vs70.40±13.09, p=0.001) components of the SF-36 question-naire between the two (PMMF and ALTFF) groups. The rest of the components did not show a significant difference ( Table 3).

**Table 2:**
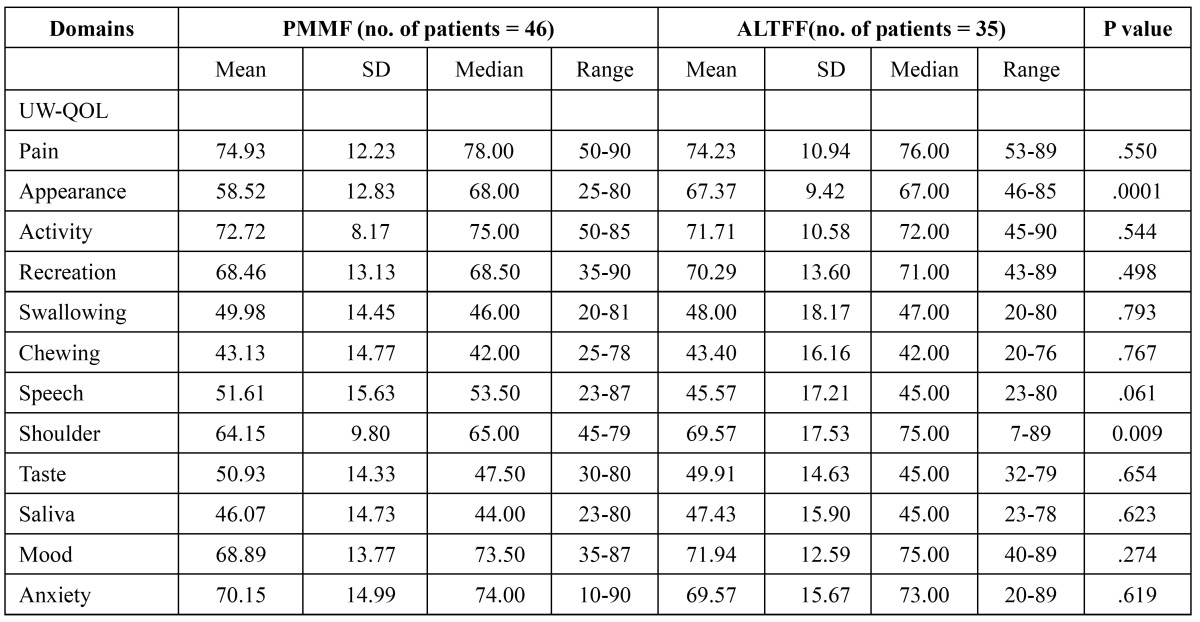
Means of scores of items and scales of UW-QOL questionnaire.

**Table 3:**
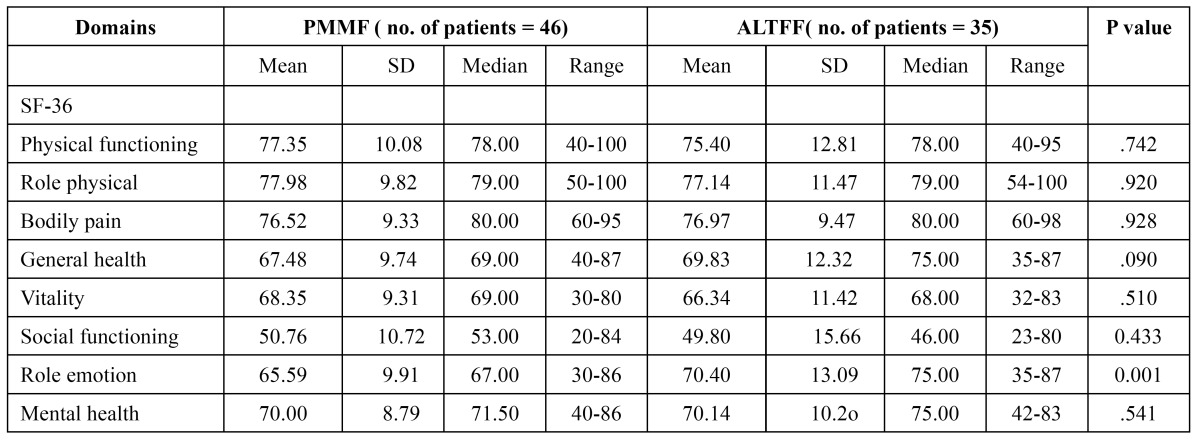
Means of scores of items and scales of MOS SF-36 questionnaire.

## Discussion

This cross-sectional study was compared the QOL of patients in Chinese population who underwent PMMF or ALTFF reconstruction after oral cavity cancer extirpation. Presently, it is generally acknowledged that free tissue transfer with micro-vascular anastomosis is the favored method for reconstruction after major head and neck cancer surgery (10). The ALTFF is becoming one of the most preferred options for soft-tissue reconstruction. It helped to shift focus away from simple coverage of defects towards minimizing morbidity at the donor site, refining the flap, reducing bulk, etc. However microsurgical reconstructions are not without potential morbidities, require specialized surgical skills, and are often lengthy procedures. The PMMF provided reliable and ample vascularized soft tissue bulk with skin coverage for defects. The PMMF quickly became the flap of choice for primary reconstruction. Despite these benefits, regional pedicled flaps such as the PMMF were accompanied by a number of significant drawbacks including minimal pliability, a restrictive pedicle length, significant tissue bulk-limiting reconstruction of 3-dimensional defects, and long-term side effects of contracture with poor aesthetics (11). To the best of our knowledge, this study is the largest series to compare the differences between patients who have undergone PMMF and ALTFF reconstruction after ablation of oral cavity cancer.

There were no signiﬁcant differences between the PMMF and ALTFF groups for age (p=0.961), primary tumor site (p=0.717), T-stage (p=0.971) and treatment (p=0.497). However, there was significant difference in gender (P<0.005) between the two groups. Several previous studies found no significant difference in the gender distribution between free flap and PMMF (12,13). However, there was a higher proportion of female patients who underwent free flap reconstruction in the current study. This could be explanation might be presumed greater importance placed on cosmetic outcome (deformity of breast) among female patients resulting in a preference for ALTFF in female. All female patients in our study selected ALTFF. This was similar to that reported by Hsing et al. (14).

We found that patients who received reconstruction with ALTFF had a longer operative duration when compared with those who were reconstructed with PMMF (475 ± 128 vs 565 ± 107 min), which was a similar finding to that reported in previous studies (14,15).The need for microvascular anastomosis is may be the main reason for the longer duration of procedure.

The head and neck speciﬁc questionnaire was able to better demonstrate the changes in quality of life due to surgery. Many scholars have chosen to use the UWQOL questionnaire (9). The UW-QOL measure was chosen as the head and neck specific questionnaire because it is short and easy for patients to complete themselves, thus making it ideal in a busy outpatient setting. We found that patients who had undergone reconstruction with the ALTFF procedure had a higher score in the appearance domain when compared with those who had undergone reconstruction with PMMF. This is may be due to the donor site scar of PMMF which more likely to be exposed. However, the ALTFF’ donor site scar was closed and hidden, allowing patients to easily accept donor site morbidity. We also found that the average score in the shoulder domain from the PMMF group was worse than that of the ALTFF group. Some study showed that PMMF not only reduced the range of motion but also reduced the strength across more than one domain (16). This could explain why the average score in the shoulder domain in the PMMF group was worse than that of the ALTFF group.

Rogers et al. and Chin et al. in their study on importance-rating using the UW-QOL questionnaire in patients treated by primary surgery for oral cancer found that patients tended to rate speech, chewing, and swallowing as more important than the other UW-QOL domains (14,17). Our study found the same results. This results highlights the crucial impact of the capacity to communicate and eat on patients’ overall sense of well-being. Also we found that there were no significant differences between the two groups for the speech, swallowing, chewing, taste, saliva domains and their scores were not well. This show that Using either PMMF or ALTFF for reconstruction of oral defects after cancer resection signiﬁcantly inﬂuences a patient’s oral function.

Generic questionnaires are usually used to assess general well-being, and pay less attention to a specific disease. SF-36 was used as a generic questionnaire in our study, because it is the most widely evaluated generic patient assessed health outcome measure (8). We were not able to see any signiﬁcant differences between the PMMF and ALTFF groups regarding physical functioning, role physical, role physical, bodily pain, general health, vitality, social functioning and mental health. This shows that oral cancer surgery does seem to have an overall effect on general health. However, there was a signiﬁcant change in the role emotion components. From  Table 3, we can see that the PMMF patients score lower in the role emotion (65.59± 9.91vs70.40±13.09, p=0.001) and domains. We believe that the donor site scar affected patients’ normal social activities and social interaction, so it brings a great deal of distress to patients. Therefore, for PMMF, donor-site morbidity such as scar appearance and aesthetics have typically become important social and emotion issues.

Some studies have suggested that after radiotherapy, weight, salivary function and physical function were significantly reduced and that swallowing, coughing, and dry mouth symptoms increased (18,19). Previous studies have shown that adjuvant radiotherapy, compared with operation alone, results in the greatest functional deficit, resulting in persistent problems with disfigurement, chewing and swallowing (18). However, our study did not collect these data, no comparison could be made.

There were several limitations in our study. First, the sample size was small and may not have had sufficient power to find more valuable results. Second, this was not a randomized study. Selection bias inevitably existed. Third, this study only described oral cancer of the study population at one point in time, and thus could not fully assess the impact of patients’ QOL over the whole period of post operation. Some patients’ QOL results may have been affected by chemotherapy or radiotherapy treatment that may last 3–6 months after completion of treatment.

## Conclusion

QOL is important when assessing the outcome of treatment for patients with head and neck cancer. Using PMMF or ALTFF for reconstruction of oral cavity defects after cancer resection signiﬁcantly inﬂuences a patient’s QOL. Patients reconstructed with ALTFF had a better appearance and better shoulder function as well as better role emotion when compared to those patients reconstructed with PMMF, which should be considered for future surgical planning.
